# Accreditation of a molecular HIV diagnostic laboratory following the Strengthening Laboratory Management Towards Accreditation (SLMTA)-Stepwise Laboratory Quality Improvement Process Towards Accreditation (SLIPTA) approach in Kenya: an implementation science study

**DOI:** 10.11604/pamj.2023.46.60.39549

**Published:** 2023-10-17

**Authors:** Joy Mwende Ndunda, James Sitati, Mary Inziani, Rebecca Loraine Achieng, Janepher Achieng, Laurie Kennedy, Cynthia Kademba, Agnes Wanjiru, Videlis Nduba, Carolyne Ndila, Matilu Mwau

**Affiliations:** 1 Kenya Medical Research Institute, Mbagathi Road Off Mbagathi Way, Nairobi, Kenya

**Keywords:** Viral load, HIV, accreditation, SLMTA, SLIPTA, molecular laboratory

## Abstract

**Introduction:**

accreditation is the most effective approach to ensure the quality of services. Laboratory performance can be evaluated using the World Health Organization (WHO)-SLIPTA checklist, which checks a laboratory´s compliance with ISO 15189 on a five-star score scale and improved using the SLMTA approach. Compliance is assessed by an external body and can result in accreditation. In this paper, we describe the steps taken by the Kenya Medical Research Institute (KEMRI) HIV Laboratory, Alupe, a resource-limited public entity, towards accreditation, and discuss the lessons learned.

**Methods:**

the laboratory adopted a SLMTA-SLIPTA approach that included targeted mentorship, on-site workshops, and training. Mentorship-based interventions were used to establish a robust quality management system. Targeted mentorship, on-site workshops, and training were conducted between September 2015 and July 2016. Audits used the SLIPTA checklist to detect gaps in 12 quality system essentials. Performance indicators including turnaround time, external quality assurance, sample rejection rates, and corrective actions were tracked. An external assessment by the national accreditation body was conducted between September 2016 and November 2016.

**Results:**

training and mentorship-based interventions were successfully conducted. Quality management systems aligned with ISO 15189 were established. Baseline, midterm, and exit audits yielded scores of 47%, 75%, and 94% respectively. Early infant diagnosis external quality assurance scores were 100% in 2014-2016, while average viral load scores were at 60%, 70% and 90% during the same period. Turnaround time from September 2015 surpassed the 80% target. Accreditation was awarded in March 2017.

**Conclusion:**

the SLMTA-SLIPTA approach is suitable for quality improvement in resource-limited laboratories.

## Introduction

In order to achieve the joint United Nations program on HIV/AIDS (UNAIDS) 95-95-95 goals, widespread access TO VIRAL LOAD (VL) testing and early infant diagnosis (EID) is necessary [1,2]. Laboratory services are essential to effective diagnosis, treatment and monitoring of patients infected with HIV and other infectious diseases. In Kenya, VL and EID services are provided by a network of high throughput reference laboratories [3]. Since the laboratories test a large number of samples, the potential risk of systematic errors is high and can undermine confidence in these services.

High-quality laboratory testing is critical for patient care, disease prevention, and surveillance [4]. Provision of quality services can be partially guaranteed through setting up quality management systems (QMS), a set of policies, processes and procedures that direct and control an organization with regard to quality [5]. Quality management systems (QMS) is composed of twelve essential interconnected building blocks that ensure processes are carried out in a systematic manner to allow for continuous improvement, meet regulatory requirements, and achieve customer satisfaction [6]. International Organization for Standardization (ISO) 15189 assesses the competence of QMS within the laboratory, providing a framework for increased analytical quality and verifying that laboratories are not deviating from quality and competency standards [7].

Achievement of ISO 15189 accreditation demonstrates competency in providing quality services and technical competency in conducting testing [6]. The importance of establishing QMS and achieving accreditation cannot be understated. However, it is a costly undertaking especially in developing countries where laboratory infrastructure and personnel are already affected by lack of resources and prioritization. Accreditation requires leadership, time, attention, resources and continuous commitment to evaluation and improvement [8]. Fulfilling the requirements of international and/or regional laboratory accreditation schemes has proven a challenge within the sub-Saharan region due to the financial implications on an already burdened system [9].

Implementation of quality systems and successful accreditation of laboratories in low and middle countries has been achieved through combining the Strengthening Laboratory Management Towards Accreditation (SLMTA) task-based program and the WHO Stepwise Laboratory Quality Improvement Process Towards Accreditation (SLIPTA), which is a stepwise accreditation preparedness program [10]. SLMTA is a hands-on training program aimed at effecting tangible laboratory improvements in developing countries [11]. It includes a series of three workshops that are supplemented by assigned improvement projects and supportive site visits or mentoring [12]. Laboratory performance is evaluated using the WHO Regional Office for Africa (WHO/AFRO) SLIPTA checklist which checks a laboratory´s compliance with ISO 15189 on a five-star score scale [13].

In 2010, Kenya adopted the SLMTA program to improve overall quality of laboratory services. The EID/VL network in Kenya has 10 reference laboratories, out of which three were already accredited by 2010 [14]. It was determined that the remaining seven laboratories should begin the process of achieving accreditation status. The Clinical and Laboratory Standards Institute (CLSI) was contracted to offer supplementary training and focused mentorship to these 6 laboratories. The HIV Laboratory, Alupe is one of the laboratories at the Kenya Medical Research Institute (KEMRI). It was established in 2010 to provide HIV laboratory diagnostic and monitoring services and is part of the EID/VL network. By 2015, the laboratory was not accredited; however, like the rest of KEMRI, it had adopted ISO 9001: 2005 and ISO 15189: 2012 guidelines to setup policies and procedures. Quality performance was monitored using the quality indicators which included enrolment in external quality assurance (EQA), laboratory turnaround time and sample rejections. However, the QMS were insufficiently robust to assure quality laboratory services.

In this paper, we describe the experiences, challenges faced and lessons learned by the KEMRI HIV laboratory, Alupe during its journey to set up QMS and obtain accreditation.

**Objective:** to outline the progress towards accreditation through implementation of the SLMTA-SLIPTA approach at KEMRI HIV Laboratory, Alupe.

## Methods

**Study design:** this was an implementation science study; qualitative data was collected through longitudinal observation.

**Study setting:** the KEMRI HIV Laboratory, Alupe is located in Western Kenya, Busia County along Malaba Road. It supports research studies and also provides diagnostic services in support of the national HIV program. The accreditation process began in September 2015 and was concluded in March 2017. The study obtained data from patient samples collected at comprehensive care clinics in various health facilities in Western Kenya networked to KEMRI HIV Laboratory, Alupe for routine VL and EID testing. This data covered the September 2015-March 2017 period when mentorship was ongoing.

**Participants:** the study was implemented by laboratory scientists and technicians.

**Variables:** variables collected were Internal audit scores, turnaround time, EQA performance, rejection rates, and corrective actions.

**Data sources/measurement:** data was collected by observation. Turnaround time and rejection rates were calculated using the laboratory information management system (LIMS). Audits were assessed using the SLIPTA scoring system based on weighted marks out of a total of 258 points and the star rating was as follows: 0-142 points: 0 stars, 143-165 points: 1 star, 166-191 points: 2 stars, 192-217 points: 3 stars, 218-243 points: 4 stars and 244-258 points: 5 stars. EQA performance was collected from EQA reports and corrective actions were collated from audit reports and corrective action forms.

**Bias:** samples of borderline quality were rejected or accepted subjectively. This may have led to bias in the rejection rates.

**Study size:** this study involves a performance matrix for just one laboratory.

**Quantitative variables:** turnaround time, rejection rates, and EQA results.

**Statistical methods:** simple descriptive statistics were used to calculate turnaround time and rejection rates. Data was presented in graphs and tables.

**Inception and planning:** in a meeting held in August 2015, SLMTA in-country mentors from CLSI and the laboratory management jointly agreed to initiate steps towards accreditation, using the SLMTA program approach and the WHO SLIPTA checklist. The proposed mentorship included three workshops spaced throughout the mentorship sessions and improvement projects to effect immediate and measurable laboratory improvements. Regular supervisory visits and on-site training were proposed. The training was focused on targeting each Quality system essential (QSE) and undertaking improvement projects aimed at addressing gaps.

**Internal audits:** it was agreed in the planning meeting that audits would be carried out in three phases, baseline audit in September 2015, mid-term audit in January 2016, and exit audit in July 2016. These were conducted using the SLIPTA checklist to assess strengths, weaknesses, and progress made. The checklist scoring system was based on weighted marks out of a total of 258 points and the star rating was as follows: 0-142 points: 0 stars, 143-165 points: 1 star, 166-191 points: 2 stars, 192-217 points: 3 stars, 218-243 points: 4 stars and 244-258 points: 5 stars.

Ten mentorship sessions were conducted between September 2015 and July 2016. Each session was planned to run over a period of two weeks. Various activities were proposed for the mentorship and internal audit process. First, a baseline audit was conducted to establish the status of the laboratory in terms of QMS implementation. This would then be followed by a two-week mentorship session to address the existing gaps. Over the next two weeks, an action plan was developed, and implementation was conducted over a two-week period. These steps were repeated for a period of 5 sessions prior to a mid-term audit.

The mid-term audit was planned to measure the overall progress from the mentorship sessions where QSE targets would be reviewed and an action plan generated. Following this audit, a series of targeted sessions, workshops, and trainings were planned to address the gaps identified in the audit. The next five sessions focused on further QMS implementation culminating in an exit audit.

**Performance of quality indicators:** quality performance was monitored using quality indicators including turnaround time (TAT), external quality assurance, sample rejection rates, and corrective actions.

**Turnaround time (TAT):** using the national guidelines of turnaround time (TAT) of 5 days for EID and 10 days for VL, the laboratory monitored the number of samples that had attained this requirement over a period of one year (September 2015 to August 2016). The percentage of the number of samples that had met the national TAT requirements was calculated as a total number of samples meeting TAT against samples received. Using the laboratory set guidelines that required at least 80% of the samples to meet TAT, the calculated percentage was compared against this set threshold.

**External quality assurance (EQA):** the laboratory receives external quality assurance (EQA) panels from the Global AIDS Program (GAP)-Centers for Disease Control and Prevention (CDC) proficiency testing program for both VL and EID in two cycles. Cycle 1 panels were received within the first quarter of the year and cycle 2 panels within the third quarter of the year. The acceptable performance was defined by any EQA panel scoring at least 80% in each cycle.

**Sample rejection rates:** rejections were monitored on a monthly basis for a period of 21 months. Rejection criteria for viral load plasma samples included hemolysis, sample identification (ID) mismatch, sample clots, use of expired sample collection tubes, wrong sample type, compromised temperature during transportation, missing request form, and insufficient sample volume. Rejection criteria for EID samples included missing request forms, sample clots, improper packaging, and insufficient samples. The rejection rate was calculated as a percentage of the total samples rejected over the total samples received. A 2% acceptable sample rejection limit was set.

**Corrective actions:** corrective actions were evaluated based on corrective action forms and audit reports. The baseline audit was performed in September 2015, a midterm audit was conducted in January 2016, and the exit audit in July 2016. The laboratory was assessed based on the 12 quality essentials and this involved evaluating the requirements of the twelve quality essentials based on ISO 15189: 2012.

**External audits:** accreditation covers QMS set-up and implementation and the technical competence to carry out diagnostics within the scope of the accreditation. The Kenya National Accreditation Service (KENAS), which is the national body mandated to oversee the accreditation of laboratories to the ISO 15189 standard, was contracted by KEMRI to provide audit services at a cost. An assessment was performed between September 2016 and November 2016 to evaluate the compliance of the laboratory to ISO 15189: 2012 using KENAS checklist.

## Results

**Participants:** a total of 15 laboratory scientists and technicians were involved in this study.

**Descriptive data:** data on internal audits, turnaround time, rejection rates, EQA performance, and corrective actions was collected and described.

**Outcome Data:** the primary outcome was laboratory accreditation. Secondary outcomes included scores in audits and improvements in quality indicators.

### Main results

**Inception and planning:** we commenced this process successfully in September 2015 beginning with targeted training on the 12 QSE conducted on-site facilitated by SLMTA-trained mentors. Each intervention was geared towards aligning the laboratory processes to the ISO 15189 standard and establishing a robust QMS system. A comprehensive list of QSE, mentorship-based interventions, and outcomes are presented in Table 1.

**Table 1:** quality systems essentials (QSE) based-mentorship interventions and outcomes used in quality management systems (QMS) implementation and monitoring at KEMRI HIV Laboratory, Alupe, 2015-2016

QSE	Mentorship interventions	Outcomes
Documents and records	Generation of all documents aligning with the ISO 15189 standard	Standard operating procedures, manuals, forms
Management review meeting	Review of management responsibilities for laboratory quality management system, on relationship between laboratory audits, customer satisfaction, quality indicators, corrective actions, and feedback loops	Generating management review schedules and incorporating them into quality plans
Organization and personnel	Training on laboratory organization, staffing matrix, continuous education, competency assessments, appraisals, and communication for quality services	Generating staff personnel files and initiating competency assessments
Client and customer care management	Initiating customer surveys, complaint registers and communication networks to get feedback from clients	Collecting data from customer surveys, complaints, and overall client feedback
Equipment	Mentorship on maintenance of equipment files, method validation and verification, routine equipment maintenance	Drafting service contract agreements with machine manufacturers and maintaining an equipment inventory and maintenance log
Evaluation and audits	Training on conducting internal audits using SLIPTA checklist	Drafting an annual audit schedule
Purchasing and inventory	Initiating inventory controls to track supplies received at the laboratory	Introducing reorder levels, inventory logs
Process control	Review of external quality assessments and internal quality control procedures, identification of barriers, outcomes, root cause analysis, and corrective actions for unacceptable external quality assessment results	Documenting trends in EQA, and internal quality controls
Information management	Refresher on reporting of validated laboratory results and use of verified laboratory information system to manage laboratory reports	Developing procedures on information management system verification
Identification of non-conformities, corrective action, and preventive action	Mentorship on how to conduct internal audits, investigating the root cause, and appropriate corrective actions	Documenting trends in non-conforming events, developing documents for recording non-conformities and their corrective and preventive actions
Occurrence management and process improvement	Training on quality indicator monitoring and implementation of the tools	Identify and track quality indicators at different time intervals
Facilities and biosafety	Guiding laboratory staff on safety requirements and various safety training	Track any adverse events within the laboratory, development of manuals and procedures, adoption of national waste management policies

EQA: external quality assurance; HIV: human immunodeficiency virus; ISO: International Organization for Standardization; KEMRI: Kenya Medical Research Institute; SLIPTA: Stepwise Laboratory Quality Improvement Process Towards Accreditation; QMS: quality management systems; QSE: quality systems essentials

**Figure 1:**
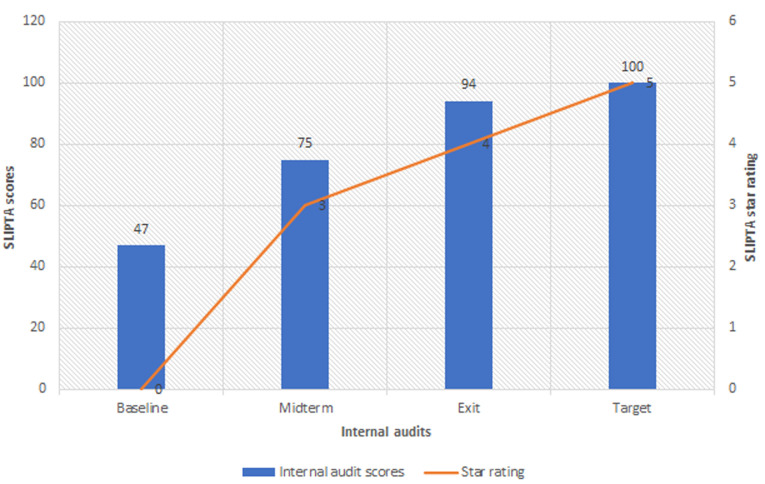
WHO Regional Office for Africa (WHO/AFRO) SLIPTA scores and star rating, KEMRI Alupe Laboratory

**Internal audits:** the lab scored zero stars (47%) at the baseline audit conducted in October 2015, three stars (75%) at the midterm in January 2016, and four stars (94%) at the exit in July 2016 (Figure 1).

The gaps that were identified, improvement projects, monitoring indicators, outcomes, and time of closure are all presented in Table 2.

**Table 2:** quality systems essentials (QSE) Gaps, improvement projects and outcomes during quality management systems (QMS) implementation using SLMTA-SLIPTA approach at KEMRI HIV Laboratory Alupe, 2015-2016

Quality essential	Gap identified	Improvement project	Monitoring indicator	Outcome	Time of closure
Documents and records	No legal entity, no quality manual	Follow up with KEMRI through the PI; develop quality policy manual	Availability of gazette KEMRI ACT; quality policy manual in place	Obtained the Legal entity Implementation of approved Quality policy	Exit Midterm
Management reviews and management responsibilities	No management review meetings and schedules in place	Develop a quality plan/schedule	Reports of MRM on file	Implementation of developed quality plan	Midterm
Organization and personnel	No designated QA officers	QA officer appointed	Appointment letter issued and JD specified	Active QA office	Midterm
Client management and customer service	No evidence of client training by qualified staff; no customer satisfaction surveys	Develop the laboratory handbook and training schedule for clients; develop a quality plan/schedule	Client training logs filed; file completed survey tools	Trained clients Satisfied customers	Exit Midterm
Equipment	Inadequate equipment installation and placement records; no evidence of QC checks after equipment repair	Develop equipment management procedure; performing QC checks after equipment repair	Filing of equipment installation and placement records; copies of QC Checks are attached to repair record and filed	Updated record for all equipment; efficient equipment operation	Exit; midterm
Evaluation and audits	No risk management plan	Develop a quality plan and risk assessment tool	Records of identified and action taken filed	Proper identification and management of risks	Exit
Purchasing and inventory	Inadequate environmental monitoring of storage areas	Develop environmental monitoring tools	Completed Environmental monitoring tools filed	Environmental monitoring of the storage area done	Midterm
Process control	No records of the selection and evaluation of referral laboratories	Develop procedure for selection and evaluation of referral laboratories	Completed referral checklist and referral laboratories list filed	Reduced service delay/ backlogs	Midterm
Information management	No evidence of LIMs selection	Follow up on LIMs selection report	LIMs selection report in the file	Accessible LIMs selection report in the laboratory	Exit
Identification of non-conformities, corrective and preventive action	No NC registers	Develop NC, CA, and PA management procedure	Completed NCs register	Updated RCA and CA	Midterm
Occurrence management and process improvement	No clearly defined quality indicators	Develop a quality indicator monitoring tool	Quality Indicators reports filed	Periodic QI monitoring	Midterm
Facilities and biosafety	No safety officer	Appoint safety officer	An appointment letter issued and JD defined	Competent safety office	Midterm

CA: corrective action; EQA: external quality assurance; HIV: human immunodeficiency virus; ISO: International Organization for Standardization; JD: job description; KEMRI: Kenya Medical Research Institute; LIMS: laboratory information management systems; MRM: management review meeting; PA: preventive action; PI: principal investigator; SLIPTA: Stepwise Laboratory Quality Improvement Process Towards Accreditation; SLMTA: Strengthening Laboratory Management Towards Accreditation; QA: quality assurance; QC: quality control; NC: non conformity; QMS: quality management systems; QSE: quality systems essentials

**Figure 2:**
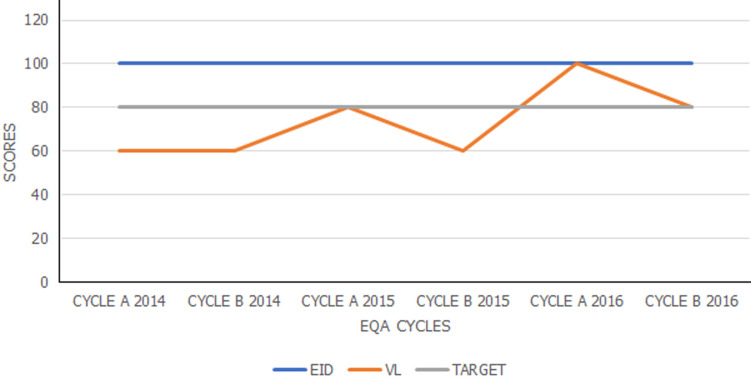
performance in early infant diagnosis (EID) and viral load (VL) external quality assurance (EQA) Indicators

**Performance in external quality assurance:** qualitative testing (EID) and quantitative testing (VL) were the two parameters chosen during the objective setting for external quality assurance (EQA). The lab scored 100% in EID EQA throughout the 6 cycles between 2014 and 2016. For VL EQA, the lab scored 60% in cycle A in 2014, 60% in cycle B in 2014, 80% in cycle A in 2015, 60% in cycle B in 2015, and 100% in cycle A in 2016, 80% in cycle B in 2016. These results are presented in Figure 2.

**Figure 3:**
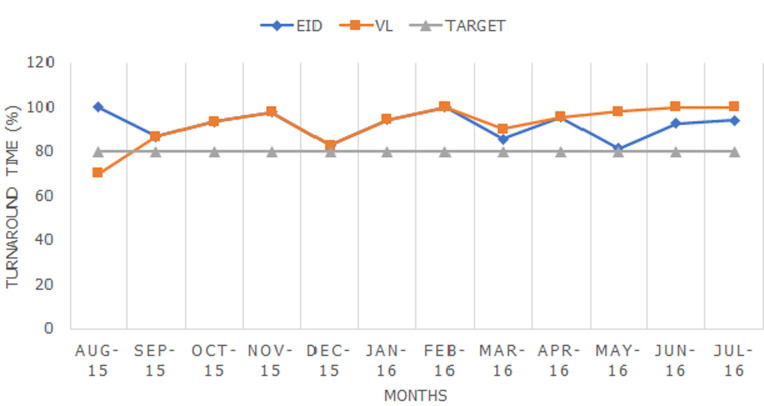
turnaround time for both early infant diagnosis (EID) and viral load (VL) tests

**Performance in laboratory turnaround time:** for EID, turnaround time (TAT) scored between 80% and 100%, whereas viral load TAT scored between 70% and 100% (Figure 3).

**Figure 4:**
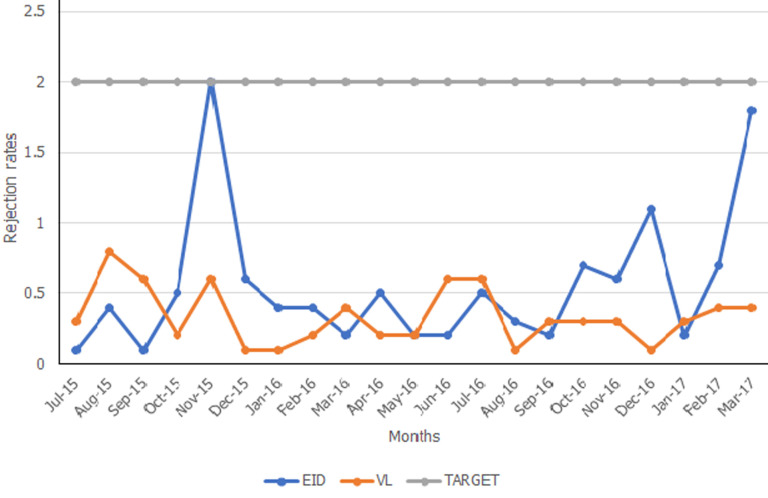
early infant diagnosis (EID) and viral load (VL) sample rejection rates

**Laboratory rejection rates:** the lab maintained a rejection rate below 2% for all the tests for the duration of the exercise. This data is shown in Figure 4.

**Figure 5:**
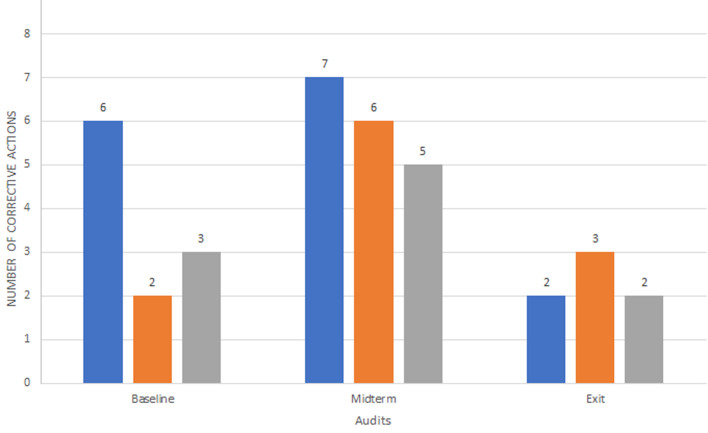
summary of corrective actions identified during baseline, midterm and exit audits

**Corrective actions:** corrective actions were evaluated and the results outlined correspond to pre-analytical, analytical, and post-analytical phases (Figure 5).

**External audits and subsequent accreditation:** a KENAS audit was successfully conducted in September 2016 and 12 non-conformities were identified; all were closed within 30 days. During the final assessment conducted in November 2016 after the submission of corrective actions from the September audit, the lab scored 96% and was deemed suitable for accreditation. Accreditation was awarded in March 2017.

## Discussion

At the baseline audit, performed in September 2015, the KEMRI HIV Laboratory, Alupe was rated at zero stars. This was despite the fact that in the year before the accreditation process started in August 2015, the laboratory had attempted to put a quality management system in place. The laboratory has been in existence since 2010, and has several performance indicators in place including EQA, TAT monitoring, and sample rejection monitoring. Worldwide, using the SLMTA approach, 84% of all laboratories scored at least 1 star [11]. The baseline survey for the KEMRI HIV Laboratory, Alupe not only indicated glaring gaps in quality system essentials but also implied that just having good intentions and an idea about QMS is not enough to provide quality services. Through the SLMTA-SLIPTA approach, and working with CLSI, the laboratory successfully developed an effective quality management system. This particular approach has also been successfully used in other countries such as Tanzania, Ethiopia [15]. At the end of the accreditation process, the laboratory scored 94% in key metrics, with a four-star rating. This score is higher than the global average of 64%, with only 13.6% of laboratories enrolled in SLIPTA reaching four and five stars by exit audit [11]. We think that strong support from the mentoring partner, as well as committed, motivated, and skilled staff, were the key ingredients in this success. Staff motivation is corroborated as a factor of success by other studies.

Sequential evaluation of the corrective actions was significant in determining the progress of QMS implementation and adherence to the ISO 15189: 2012. Corrective actions were analyzed from audit reports generated from baseline, midterm, and exit audits. The corrective actions were divided into pre-analytical, analytical, and post-analytical phases. Generally, the variation in the number of corrective actions identified across the four types of audits was contributed by the goal and the nature of each audit. At baseline, the auditors focused on the major gaps existing within the laboratory, and therefore a smaller number of corrective actions were addressed compared to the midterm audit. Besides, some areas in the SLIPTA checklist had not been established at the time of baseline audit and therefore there were fewer corrective actions raised in these particular areas, unlike the midterm audit where all the parameters of the checklist had been established by the laboratory and were assessed. The exit audit recorded a lower number of corrective actions as most of those that had been raised during the mid-term audit had been closed.

Whereas EQA for EID consistently returned excellent outcomes, the performance of VL fluctuated from cycle to cycle. This could be attributed to equipment downtime, faulty backups, and VL EQA panel limitations. VL EQA panels are delivered in small volumes that are insufficient for retesting after failure due to equipment downtime. Other studies evaluated their EQA performance before and after accreditation and noted significant improvement in performance after accreditation [16]. Viral load (VL) turnaround time, from sample reception in the lab to release of results, showed remarkable improvement from 60% in August 2015 to 100% in 2016. The poor performance in 2015 could be attributed to a surge in the number of samples being received in the laboratory without a proportional increase in human resources, stock outs of consumables and reagents, and machine breakdown. This is a common challenge in high throughput labs and requires close collaboration between the laboratory and key stakeholders. Similar findings regarding improvement in TAT were seen in a number of studies with some studies having TAT percentages at 92% [17].

Throughout the mentorship period, rejection rates did not exceed the set limit of 2%. This excellent rate was attributed to the development of the sample collection manual and its distribution to facilities served by the laboratory. Introduction of facility training also played a role in this reduction. However, the laboratory had no direct control of the quality of the samples received from the field and could not sustain targeted training due to resource limitations. Sample rejection rate therefore looks like an imperfect quality indicator in resource-limited laboratories. It may be much more appropriate in reference laboratories that are able to institute and maintain sample collection training for the health facilities they serve. Rejection rates have been shown in similar studies to significantly reduce after accreditation [14,17].

Fulfilling the requirements of international and/or regional laboratory accreditation schemes has proven to be a challenge due to the high costs of closing gaps [18]. Most existing gaps required only a little investment in resources and were therefore easily closed by the midterm audit. Other gaps were harder to close, requiring significant investment. For instance, the laboratory initially could not show evidence of client training. Client training required financial resources the laboratory did not have; these were eventually provided by partners.

The laboratory was not an independent legal identity, rather, it is part of a legal entity. The audit tools are designed for institutions with legal identities. This lack of individual identity was considered a gap and took significant time and effort to be resolved.

Gaps identified under equipment QSE included inadequate equipment installation and placement records, and lack of instrument calibration. The placement of machines is under the control of the ministry of health (MOH); this lack of control by the laboratory causes significant challenges with procurement and maintenance. Even the selection, purchasing, and verification of LIMS is under MOH. The tools used by auditors need to be customized to cater to such peculiarities, especially within public laboratories. Inadequate waste management and lack of evidence of space evaluation were the main gaps identified in facilities and safety QSE. The former required the installation of an incinerator while the latter needed several interventions including infrastructural changes. All these required management support and significant resources.

Achieving and maintaining accreditation has significant monetary implications and for that reason, laboratories in Africa that have received accreditation have tended to be privately funded or partner-supported [19-21]. A major contributor to that cost was external audits, which turned out to be even more costly than many budget items for service delivery. On further analysis, it was realized that KENAS provides auditors from a central location, and all expenses are met by the laboratories. This expensive and inefficient model, can cause public laboratories pecuniary embarrassment, and they can benefit from decentralization of audit services.

**Lessons learned:** from the foregoing, it is clear that laboratories ought to enroll and participate in established QMS systems. Even then, not all that do so achieve excellence. In our experience, it was essential to have resources, trained and motivated staff, accreditation champions, and buy-in from top-level management. Many of the gaps in the quality system essentials were attributed to a lack of resources. The gaps were addressed through the provision of financial resources either by the mentoring partner or very rarely, by KEMRI. The reluctance of the parent institution to invest in accreditation might have arisen from a lack of political buy-in and strategic planning from the outset. The laboratory team found it especially difficult to convince top-level management that investing in accreditation can lead to significant cost-savings, particularly when such discussions are initiated midway through the process. Public laboratories, which are particularly vulnerable, would do well to introduce accreditation within the key results areas of institutional strategic plans.

A second lesson learned is that to obtain accreditation in a public laboratory, staff must be willing to provide creative solutions and to work extra hard including outside of office hours. Staff motivation and management involvement are critical for success in accreditation.

**Limitations:** the single most important limitation in this study was that the laboratory is a public asset managed through government bureaucratic systems. In this environment, activities that involve finances or procurement are often slow and sometimes fail. This makes our findings hard to generalize across all laboratories. Secondly, we were unable to quantify the costs associated with implementing the SLMTA-SLIPTA approach. This was because the laboratory was a public facility and several costs were invisible to the implementing team.

## Conclusion

The SLMTA-SLIPTA approach is suitable in the accreditation of resource-limited laboratories. It is recommended that the approach is modified to be relevant to laboratories that are only part of a legal entity. The auditor´s responsibilities of identifying areas of non-conformity and providing onsite technical assistance are a “game-changer” unique to this approach. However, the annual subscription fees exceed the ability of many facilities and this is a key reason accreditation is a pipe dream for most resource-limited laboratories. With sustained input from the management, laboratory staff, mentors, and collaborators, using the stepwise improvement process, any laboratory can improve on its quality systems and implement QMS that is compliant with the standards of ISO 15189: 2012.

### What is known about this topic

- *Accreditation is the most effective approach to assure quality of all laboratory services;*
- *Getting accreditation is especially difficult for poorly resourced laboratories and only 13.6% of laboratories enrolled in SLIPTA reach four and five stars by exit audit*.

### What this study adds

- *In this study, our findings suggest that the SLMTA-SLIPTA approach needs to be modified to be relevant to laboratories that are only part of a legal entity, or that are public assets managed through government bureaucratic systems*.
